# Web-Based Mindfulness Interventions for Mental Health Treatment: Systematic Review and Meta-Analysis

**DOI:** 10.2196/10278

**Published:** 2018-09-25

**Authors:** Julia Sevilla-Llewellyn-Jones, Olga Santesteban-Echarri, Ingrid Pryor, Patrick McGorry, Mario Alvarez-Jimenez

**Affiliations:** 1 Institute of Psychiatry and Mental Health Health Research Institute (IdISSC). Hospital Clínico San Carlos Madrid Spain; 2 Mental Health Department Clinico Virgen de la Victoria Hospital Málaga Spain; 3 Orygen The National Centre of Excellence in Youth Mental Health Melbourne Australia; 4 Faculty of Psychology Malaga University Malaga Spain; 5 Centre for Youth Mental Health The University of Melbourne Melbourne Australia; 6 Faculty of Health Sciences Universidad Internacional de la Rioja (UNIR) Madrid Spain

**Keywords:** mindfulness, anxiety disorder, depressive disorder, internetinternet-based, treatment, meta-analysis, mental health., systematic review

## Abstract

**Background:**

Web-based mindfulness interventions are increasingly delivered through the internet to treat mental health conditions.

**Objective:**

The objective of this study was to determine the effectiveness of web-based mindfulness interventions in clinical mental health populations. Secondary aims were to explore the impact of study variables on the effectiveness of web-based mindfulness interventions.

**Methods:**

We performed a systematic review and meta-analysis of studies investigating the effects of web-based mindfulness interventions on clinical populations.

**Results:**

The search strategy yielded 12 eligible studies. Web-based mindfulness interventions were effective in reducing depression in the total clinical sample (n=656 g=−0.609, *P*=.004) and in the anxiety disorder subgroup (n=313, g=−0.651, *P*<.001), but not in the depression disorder subgroup (n=251, *P*=.18). Similarly, web-based mindfulness interventions significantly reduced anxiety in the total clinical sample (n=756, g=−0.433, *P*=.004) and the anxiety disorder subgroup (n=413, g=−0.719, *P*<.001), but not in the depression disorder group (n=251, g=−0.213, *P*=.28). Finally, web-based mindfulness interventions improved quality of life and functioning in the total sample (n=591, g=0.362, *P*=.02) in the anxiety disorder subgroup (n=370, g=0.550, *P*=.02) and mindfulness skills in the total clinical sample (n=251, g=0.724, *P*<.001).

**Conclusions:**

Results support the effectiveness of web-based mindfulness interventions in reducing depression and anxiety and in enhancing quality of life and mindfulness skills, particularly in those with clinical anxiety. Results should be interpreted with caution given the high heterogeneity of web-based mindfulness interventions and the low number of studies included.

## Introduction

In most countries reporting sufficient data, at some point in their lives, over a third of people meet the criteria for being diagnosed with a mental health disorder [1,2]. The most prevalent psychological disorders are anxiety disorders, followed by mood disorders, externalizing disorders such as attention deficit or hyperactivity disorder or oppositional defiant disorder, and substance use disorders [2]. The average lifetime prevalence for depression and anxiety is 11% and 16%, respectively [2,3], and they tend to co-occur [3]. Furthermore, anxiety and depression disorders have high comorbidity with suicide attempts [4]. Developing and evaluating interventions for these disorders is therefore essential.

Psychological interventions are the treatment of choice for mild to moderate mental health conditions such as depression and anxiety [5-7]. In recent years, mindfulness-based interventions (MBIs) have shown promising results [8,9], and mindfulness-based cognitive therapy (MBCT) is recommended as the treatment of choice for relapse prevention in recurrent depression [10].

Mindfulness derives from Buddhist practice and is described as an intentional and nonjudgmental awareness of the present moment [11]. MBIs are assumed to decrease distress by encouraging individuals to relate to their experiences with acceptance and compassion instead of avoidance, control, or suppression [12,13]. Mindfulness is used in various interventions, each tailored for use with specific populations. Examples include Mindfulness-Based Stress Reduction (MBSR) and MBCT tailored for various target populations. In fact, evidence indicates that MBCT is effective in preventing depressive relapse [14,15]. Other interventions, such as Acceptance and Commitment Therapy (ACT), combine principles of mindfulness and acceptance with treatment components from behavioral therapy and experiential psychotherapy [16].

Over the last few years, interest in MBI efficacy has accrued [17]. From the effectiveness of MBCT in preventing relapse in recurrent depression [18-20] to the application of MBI in other mental health conditions, such as substance use, attention deficit or hyperactivity, and anxiety and depression disorders, research has indicated efficacy [8,9,21,22]. However, two recent meta-analyses have reported conflicting results. Vøllestad, Nielsen, and Nielsen [9] have investigated the effects of MBI on anxiety disorders, reporting a large effect size on reducing anxiety symptoms (*g*=−0.83) and depressive symptoms (*g*=−0.72). On the other hand, Strauss, Cavanagh, Oliver, and Pettman [8] investigated the effects of MBI on both anxiety and depressive disorders. In contrast to Vøllestad et al’s results, Strauss et al did not find a significant effect of MBI on anxiety disorders (*P*=.09). However, MBI had a significantly large effect in reducing both depressive symptoms in those with depression (*g*=−0.73) and in depressive symptoms (*g*=−0.64) when anxiety and depressive disorders were considered together. Vøllestad et al [9] and Strauss et al’s [8] meta-analyses focused on different target populations. Furthermore, Strauss et al [8] considered only studies using a group format, while Vøllestad et al [9] included both individual and group formats. It is therefore important to investigate not only the effects of MBI but also the variables that may influence its effectiveness, including duration of treatment [23], group versus individual format [24,25] and target population [17]. Since these variables were inconsistent between Vøllestad et al [9] and Strauss et al’s [8] meta-analyses, the reasons behind their differing findings are difficult to decipher.

The previous decade has witnessed increased use of the internet, which has become more than a simple information and communication tool [26]. With increasing access to novel information and communication technologies in developed countries, a growing number of users resort to the internet for information on, and support for, mental health disorders [27]. This rapid development can be easily understood in the context of the significant advantages of online therapy, such as accessibility, low stigma, and cost effectiveness [28]. Two meta-analyses have shown that psychological interventions delivered via smartphone devices can reduce anxiety and depression symptoms [29,30]. In fact, the National Institute for Health and Care Excellence guidelines consider online cognitive behavioral therapy a first-line treatment for depression and anxiety [31]. Likewise, web-based mindfulness interventions (WMIs) have been developed with promising results [32]. WMIs’ potential advantages include reductions both in service costs and demand on mindfulness-trained therapists [33].

WMIs have been designed and applied to healthy participants [34] and individuals with physical illness, such as tinnitus [35], and mental health disorders, such as anxiety or depressive disorders [36,37]. To our knowledge, only one meta-analysis has examined the effectiveness of WMIs in clinical (physical and mental illness) and nonclinical populations [32]. This meta-analysis reported moderate, but significant beneficial impact of WMIs on depression (*g*=0.29) and anxiety (*g*=0.22) outcomes. While this meta-analysis had several strengths, such as including only online randomized controlled trials, it combined people with and without mental health disorders, with no separate analysis of WMIs’ impact on different populations. The effectiveness of MBI has been shown to differ among target populations [17]. Therefore, it is important to determine the effects of WMIs in clinical contexts and also whether MBI is more effective for various clinical conditions such as anxiety and depression.

The primary aim of this study was to update, systematically compile, and analyze the effectiveness of WMIs in patients with a diagnosed mental health disorder. Secondary aims were to explore whether study variables, including participant characteristics, type of control group, and the design and implementation of the intervention had an impact on the effect of WMIs on this population.

## Methods

This review was conducted in line with the Preferred Reporting Items for Systematic Reviews and Meta-Analyses statement (available upon request) [38].

### Data Sources

A systematic search of published studies was performed using the following databases: PubMed, PsycInfo, Web of Science, and Scopus, from inception to March 2018. No restrictions were applied for languages. The abstracts, titles, and keywords of studies were searched using combinations of the following terms: (computer OR cyber OR electronic OR email OR e-mail OR internet OR net OR online OR virtual OR Web OR www OR “social media” OR “social network” OR blog OR forum OR mobile OR smartphone) AND (mindfulness OR self-compassion* OR “compassion-based” OR “acceptance and commitment therapy” OR “acceptance-based” OR “loving kindness” OR “person-based cognitive therapy”). Additional articles were identified by hand searching references of retrieved articles and relevant reviews.

### Study Selection

To be included in this meta-analysis, studies must have involved participants with a diagnosed mental health condition using either Diagnostic and Statistical Manual of Mental Disorders [39] or International classification of Diseases [40] criteria.

WMIs were defined as Web-based interventions enabling patient-to-expert communication or internet psychoeducation or therapy. Mobile-based interventions were defined as interventions delivered via mobile phones using short message service (SMS) text messaging, multimedia messaging service, or Web apps. Given the field’s early state, we considered WMIs as any intervention that incorporated mindfulness either as a therapy (mindfulness-only therapies) or as a main but not the only component of a therapy (mindfulness integrative therapies). The latter definition includes therapies such as acceptance and commitment therapy [16] or acceptance-based cognitive behavior therapy [41].

Studies investigating traditional face-to-face therapy, delivered via teleconference, mobile phone, audiotape, or CD, or that comprised of only downloading a manual or audio file were excluded [42]. Studies that examined online interventions using mindfulness as a minor component of an eclectic therapy, such as using mindfulness as a relaxation exercise, were not included. We also excluded studies investigating the effects of WMIs on anxiety and depression symptoms in nonclinical samples or in samples with somatic disorders or where diagnosis could not be established. In addition, we did not include studies that used WMIs as an adjunct of face-to-face treatment. Finally, we excluded poster presentations and book chapters.

One reviewer (JSLJ) screened all abstracts to determine initial eligibility. Potentially relevant papers were retrieved for detailed examination. Two reviewers (JSLJ and OSE) then independently assessed the retrieved articles. Any disagreements were resolved through discussion by JSLJ, OSE, and MAJ—all clinical psychologists instructed in mindfulness. If necessary, authors were contacted to determine eligibility against inclusion criteria.

### Data Extraction and Analysis

Two reviewers (JSLJ and OSE) independently extracted relevant data from selected studies, as follows:

1. *Characteristics of the study and participants*: author; year of publication; diagnosis and diagnostic criteria; number of participants; gender; mean age; follow-up time in weeks; control group details, including type and duration of treatment for the control group (if applicable).
2. *Characteristics of the intervention*: type of intervention; length of treatment; material used (videos, email, phone, SMS text messaging, and presentations); treatment schedule (daily or weekly); setting (computer- or phone-based); assigned home tasks (if applicable); contact with therapist (if applicable).
3. *Intervention evaluation, dropout rates, and associated variables:* adherence; users’ evaluations of usability, attractiveness, and helpfulness of the intervention; dropout rates; variables associated with the use of and engagement with the intervention; adverse events and safety of the intervention.

### Assessment of Methodological Quality Procedures

Two reviewers (JSLJ and OSE) independently assessed the methodological quality of each of the studies included. For controlled studies, methodological quality was assessed using the Cochrane Collaboration “risk of bias” tool [43]. For uncontrolled studies, we assessed the following criteria: blinding to study design or purpose and incomplete outcome data [42]. We also assessed the quality of the mindfulness intervention using previously proposed criteria adapted for our purpose [17]. Specifically, we assessed whether the included studies used validated mindfulness or acceptance measures, for example, Five Faces of Mindfulness Questionnaire [44], Acceptance and Action Questionnaire [45], Kentucky Inventory Mindfulness Skills [46], Philadelphia Mindfulness Scale [47]; we also assessed the clinical and mindfulness-specific training of the therapist, if applicable, and of the developer of the online intervention.

### Data Analysis

For each comparison between treatment and control groups and for each outcome variable (depression, anxiety, quality of life and functioning, and mindfulness skills), we calculated effect size. When the same outcome was evaluated with multiple scales or domains within the same study, we retained only the most valid measure (see Results section) so that each outcome had one effect size. Effect sizes were pooled for predictors analyzed in ≥3 studies reporting data in a usable format. When not enough data was available, authors were contacted for provision of the necessary additional data.

First, pooled analyses, including all diagnoses, were performed to examine the transdiagnostic impact of WMIs. Only studies with a control group were included in this pooled analysis. For each comparison, Hedge’s *g* (a correction of Cohen *d* for small samples) was calculated using means and SDs for each outcome measure. To obtain a pre-post comparison between treatment and control groups, we used the formula *d* = (*M*_1_−*M*_0_) / *SD*_0_, where *M*_1_ and *M*_0_ are means at post- and pretest, respectively, and SD_0_ is the pretest SD. We calculated d for the treatment group (*d*_T_) and the control group (*d*_C_). Each effect size indicates, in SDs, the difference in mean between pre- and posttreatment for each group. That is, we calculated the main effect size by calculating the difference between *d*_T_ and *d*_C_. Effect sizes were estimated using Comprehensive Meta-Analysis software version 2.2 (Biostat, Englewood, NJ, USA) [48]. Finally, in order to interpret data, we followed Cohen’s [49,50] recommended benchmarks, wherein an effect size is considered small for a Hedge’s *g* of 0.20, moderate for 0.50, and large for 0.80. We used random-effects models to account for within-study error and variation in true effects across studies [51]. To further assess the robustness of our results, subgroup analyses were performed to examine the differential effects of the type of diagnosis (anxiety and depression). We did not assume a common among-study variance component across subgroups. That is, we did not pool the within-group estimates of tau-squared, as this is the option used by RevMan. Moreover, sensitivity analyses were performed to examine (1) statistical heterogeneity; (2) differences by type of therapy (ie, differences between studies using mindfulness-only therapies vs those using mindfulness integrative therapies); and (3) differences by type of control group, that is, wait list, treatment as usual (TAU), or other active control group. Finally, we analyzed the sustainability of treatment effects over time (ie, differences between comparisons of pretreatment to posttreatment vs pretreatment to follow-up) of those studies with follow-up measures. We did not include control groups in our analysis of sustainability of treatment effects because only one study [37] reported follow-up data for the control group. To test the sustainability of treatment effects, we used the formula *d* = (*M*_1_−*M*_0_) / *SD*_0_, where for each group, *M*_1_ and *M*_0_ are means at posttreatment and pretreatment, respectively, and *SD*_0_ is the pretreatment SD. That is, we calculated d for pretreatment to posttreatment (*d*_P-P_) and for pretreatment to follow-up (*d*_F-U_) and calculated the main effect size by calculating the difference between *d*_P-P_ and *d*_F-U_.

Next, we considered heterogeneity, publication bias, and sensitivity. Heterogeneity was calculated by testing the null hypothesis that the true effect size is the same in all studies using the *Q* statistic [51]. The *I*^2^statistic explains the percentage of variance in observed effects due to variance in true effects. We assessed variance of true effect sizes using *T*^2^and the SD of true effects using *T.* Publication bias was tested by entering data in a funnel graph (a plot of dispersion between study effect and a measure of study size). A symmetrical inverted distribution of the studies around the mean effect size represented in the funnel would indicate an absence of publication bias [51]. If publication bias exists, it was expected that the smallest studies would report the biggest effect sizes.

## Results

### Study Selection

**Figure 1:**
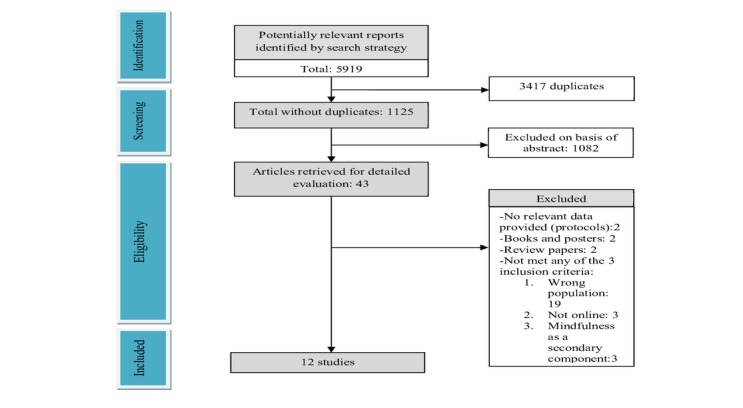
Flow chart of study retrieval and selection strategy.

Of 5919 studies retrieved, 12 were included. Figure 1 illustrates study retrieval and selection strategy.

### Characteristics of Studies and Participants

A total of 12 studies, involving 919 participants, were selected. Multimedia Appendix 1 depicts the characteristics of these studies, namely, the diagnosis criteria used to determine the eligibility, number of participants, percentage of female participants, mean age, length of treatment, length of follow-up, control group, and length of control. Patients’ mean age ranged from 33.2 (SD 10.4) to 46.6 (SD 12.9), and the majority of the sample was female. The main diagnoses were depression and anxiety disorders, frequently diagnosed by a structured interview. All studies reported posttreatment effects, and most studies reported follow-up data, had a control group, and were randomized controlled trials.

### Characteristics of Interventions

Multimedia Appendix 2 depicts the characteristics of the interventions, namely, the type of intervention, length of treatment, materials used, regularity (how often participants were required to log in), setting, the implementation structure (or lack thereof) of the intervention, assigned home task(s), and contact with therapist. Three studies tested mindfulness-only interventions [36,37,52], while four evaluated mindfulness integrative therapies. The duration of treatment varied from 3 to 12 weeks. While the majority of the interventions were flexible, computer-based, followed a modular sequence, and had assigned home tasks, the materials used and the means and frequency of contact with a therapist varied.

### Intervention Evaluation, Dropout Rates, and Associated Variables

Table 1 shows the intervention evaluation used in the selected studies. Most studies reported information about adherence and patients’ satisfaction (although each used different definitions of these variables). Dropout rates at the end of treatment varied from 0% to 38.5%. Five studies discussed associations between engagement and improved outcomes. Finally, adverse events were rarely reported.

**Table 1:** Intervention evaluation, dropout rates, and associated variables.

Study	Adherence	Users’ evaluation of usability or attractiveness or helpfulness	Dropout rates (%)	Variables associated with increased engagement and better outcomes	Adverse events
Boettcher et al, 2014 [36]	Number of completed mindfulness exercises (homework)	Satisfaction with treatment	11.11	Extensive diagnostic procedure related to adherence and therapeutic change. Clear deadline related to good outcome	Not reported
Ly et al, 2014 [37]	Number of reflections sent to therapist (homework)	Not reported	12.2	Not reported	Not reported
Carlbring et al, 2013 [53]	Number of modules finished and time spent	Not reported	0	Therapist support related to good outcome	Not reported
Kivi et al, 2014 [54]	Number of modules finished and time spent	Not reported	16.67	Extended time to complete module related to lower dropout rates. Therapist support related to better outcomes and lower dropout rates	Not reported
Murray et al, 2015 [55]	Not reported	Qualitative: content, style, negative effects and overall impressions	38.5	Not reported	Body scan meditation generated distress for one participant
Dahlin et al, 2016 [56]	Number of modules finished with homework assignment	Satisfaction with treatment or supportiveness of therapist	19.2	More pictorial information than text and extend time to complete module related to increased engagement	Not reported
Gershkovich et al, 2016 [57]	Completion of modules on weekly basis	Satisfaction with treatment or therapist or ease of use	0	Mail to remember to finish module and postpone video conference	Technical issues with videoconferences
Gershkovich et al, 2017 [58]	Number of modules finished	Satisfaction: treatment or therapist or perceived effectiveness or ease of use	31	Participants with therapist support related to increased engagement	Technical issues in videoconferences
Houghton 2008 [52]	Not reported	Not reported	27.59	Not reported	Not reported
Ivanova et al, 2016 [59]	Number of modules finished	Not reported	Not reported	Extensive use of technology related to low adherence. Personalized feedback increased the adherence to the smartphone platform in comparison to those who did not have personalized feedback	Not reported
Johansson et al, 2013 [60]	Number of modules finished	Satisfaction: amount of text or demand of the treatment or worth the effort	0	Not reported	Not reported
Strandskov et al, 2017 [61]	Not reported	Not reported	21.7	Not reported	Not reported

**Table 2:** Quality of the interventions.

Study	Use of validated mindfulness or ACT^a^ measures	Clinical training of the therapist	Mindfulness training of the therapist	Clinical training of the developer	Mindfulness training of the developer
Boettcher et al, 2014 [36]	No	Not reported	Not reported	General practitioner	Mindfulness-based cognitive therapy, mindfulness-based stress reduction, and other mindfulness training
Ly et al, 2014 [37]	AAQ-II^b^	(4th year) Clinical Psychology MSc; supervised	Not specific—as part of their training	Not applicable—platform already designed	Not reported
Carlbring et al, 2013 [53]	No	Clinical Psychology MSc; supervised	Not specific—as part of their training	Licensed psychologist	Functional contextualism and clinical behavior analysis
Kivi et al, 2014 [54]	No	Licensed psychologist or psychotherapist; supervised	Specific training for the study	Licensed psychologist	Functional contextualism and clinical behavior analysis
Murray et al, 2015 [55]	No	Not applicable	Not reported	Clinicians, costumers, and researches	Mindfulness and ACT
Dahlin et al, 2016 [56]	No	Psychologist graduate students; supervised	Not specific, but some in their training	Clinical psychologist	ACT workshops
Gershkovich et al, 2016 [57]	AAQ-II and Philadelphia Mindfulness Scale	Clinical Psychology doctoral student who received extensive training	ACT intensive training	Clinical Psychology doctoral student	ACT
Gershkovich et al, 2017 [58]	No	Clinical Psychology doctoral student who received extensive training	Not reported	Not reported	Not reported
Houghton 2008 [52]	Kentucky Inventory Mindfulness Skills	Not applicable	Not reported	Not reported	Not reported
Ivanova et al, 2016 [59]	No	Clinical Psychology MSc; supervised	Not reported	Not reported	Not reported
Johansson et al, 2013 [60]	Five Faces of Mindfulness Questionnaire	(3rd year) Clinical Psychology doctoral MSc; supervised by experienced psychotherapist	Clinical training in affect-focused psychodynamic psychotherapy	Clinical psychologist	Affect-focused psychodynamic psychotherapy
Strandskov et al, 2017 [61]	No	(4th year) Clinical Psychology MSc; supervised	Not reported	Not reported	Not reported

^a^ACT: Acceptance and Commitment Therapy.

^b^AAQ-II: Acceptance and Action Questionnaire.

### Quality of Interventions

Table 2 depicts the quality of the interventions used in the selected studies. Only four studies used a validated measure of mindfulness skills. All therapists were either graduate psychology students (master’s or doctoral level) or licensed clinical psychologists. While the training of therapists on MBI varied, the majority of developers were clinical psychologists with training in MBI.

### Methodological Quality

There were 10 randomized controlled trials and two uncontrolled studies included in this meta-analysis. The risk of selection bias was low in all studies, as an online random allocation service independent of the investigators was used. Considering the nature of the interventions, blinding of participants and personnel was not fulfilled by any study. Blinding the outcome assessment criteria was achieved by all studies, except one [54], which was the only study that did not use computer-based assessments. Finally, in both controlled and uncontrolled studies, attrition biases were assessed as low risk.

### Meta-Analytic Results

Nine outcomes were identified (stress, health, insomnia, worry, emotional processing, anxiety, depression, quality of life and functioning, and mindfulness skills), with the last four assessed in ≥3 studies.

#### Web-Based Mindfulness Interventions and Depression Outcome

**Figure 2:**
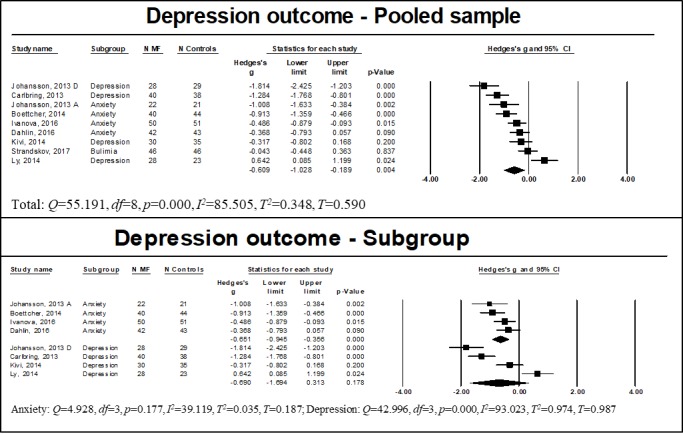
Results of depression outcome for the pooled and subgroup samples. MF: mindfulness intervention group.

Depression was examined by 11 studies, of which 5 included participants with a primary diagnosis of an anxiety disorder [36,56-59], 3 with a depressive disorder [37,53,54], 1 with both anxiety and depressive disorders [60], 1 with bipolar disorder [55], and 1 with bulimia disorder [61]. Usable data for meta-analysis of studies with a control group could be retrieved for 8 of these studies [36,37,53,54,56,59-61]. Johansson et al [60] reported data divided into two subgroups: those with a diagnosis of depression and those with a diagnosis of anxiety. We therefore considered these groups as independent samples. A summary of effect sizes is shown in Figure 2. We found significant large overall effect of WMIs on reducing depression for the pooled sample (n=656, *g*=−0.609, 95% CI −1.028 to −0.189, *P*=.004). Significant heterogeneity was noted (*Q*=55.191, *df*=8, *P<*.001, *I*^2^=85.505, *T*^2^=0.348, *T*=0.590). Subgroup analyses indicated that WMIs had a significant large effect on reducing depression among participants with a diagnosis of anxiety (n=313, *g*=−0.651, 95% CI −0.945 to −0.356, *P*<.001), with no evidence of heterogeneity (*Q*=4.928, *df*=3, *P*=.18, *I*^2^=39.119, *T*^2^=0.035, *T*=0.187). Conversely, the effect of mindfulness treatment on depression was not significant among those with a diagnosis of depression (n=251, *g*=−0.690, 95% CI −1.694 to −0.313, *P*=.19). Significant heterogeneity was noted (*Q*=42.996, *df*=3, *P*<.001, *I*^2^=93.023, *T*^2^=0.974, *T*=0.987). We performed sensitivity analyses to examine the difference between studies including mindfulness-only therapies [36,37] and those including mindfulness integrative therapies [53,54,56,59-61]. Exploratory sensitivity analysis indicated that mindfulness integrative therapies had a significant effect on reducing depression while mindfulness-only therapies did not. Furthermore, we examined differences between studies by type of control group (ie, wait list [56,59-61], TAU [54], or other active control group [36,37,53]). We found a significant effect of WMIs on only reducing depression when compared to wait list. There was not a significant difference when compared to TAU or other active control groups. For studies reporting follow-up data, analysis of the stability of treatment effects indicated that changes were stable over time (analyses are available in Multimedia Appendices 3, 4, and 5, respectively). For all outcomes, exploratory subgroup analysis should be interpreted with caution because having <5 studies per group is likely to provide an imprecise estimation [51].

#### Web-Based Mindfulness Interventions and Anxiety Outcome

**Figure 3:**
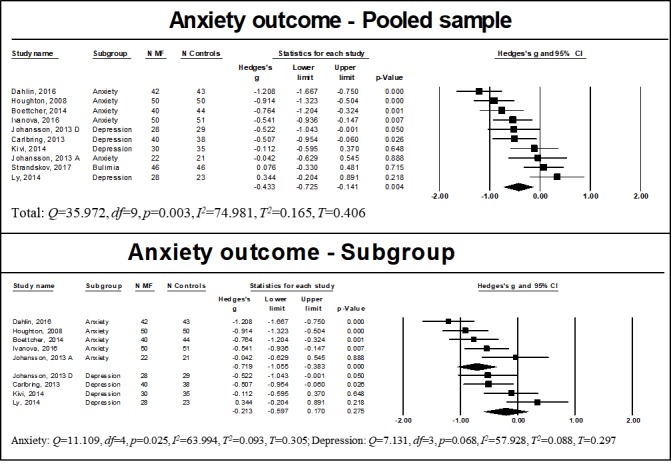
Results of anxiety outcome for the pooled and subgroup samples. MF: mindfulness intervention group.

Anxiety was examined in all studies. Of these studies, 7 included participants with a primary diagnosis of an anxiety disorder [36,52,56-60], while others included participants diagnosed with a depressive disorder in addition to anxiety [37,53,54,60], bipolar disorder [55], or bulimia [61]. Usable data of studies with a control group could be retrieved only for 9 of them [36,37,52-54,56,59-61]. Pooled effect sizes are presented in Figure 3. We found a significant moderate effect of WMIs on reducing anxiety for the pooled sample (n=756, *g*=−0.433, 95% CI −0.725 to −0.141, *P*=.004). Heterogeneity was noted (*Q*=35.972, *df*=9, *P*<.001, *I*^2^=74.981, *T*^2^=0.165, *T*=0.406). Subgroup analysis indicated that WMIs had a significant moderate effect on reducing anxiety among participants with a diagnosis of anxiety (n=413, *g*=−0.719, 95% CI −1.055 to −0.383, *P*<.001), with evidence of statistical heterogeneity (*Q*=11.109, *df*=4, *P*=.03, *I*^2^=63.994, *T*^2^=0.093, *T*=0.305). Conversely, the effect of WMIs on anxiety was not significant among those with a diagnosis of depression (n=251, *g*=−0.213, 95% CI −0.597 to −0.170, *P*=.28), with no evidence of statistical heterogeneity (*Q*=7.131, *df*=3, *P*=.07, *I*^2^=57.928, *T*^2^=0.088, *T*=0.297).

We performed sensitivity analyses to examine the difference between studies including mindfulness-only therapies [36,37,52] and those including mindfulness integrative therapies [53,54,56,59-61]. Preliminary results indicated that mindfulness integrative therapies had a significant effect on reducing anxiety, while mindfulness-only therapies did not. Mindfulness integrative therapies showed no heterogeneity, while mindfulness-only therapies showed evidence of statistical heterogeneity. Furthermore, we examined differences between studies by type of control group. We found significant effect of WMIs on reducing anxiety when compared to wait list, while there was not a significant difference when compared to TAU or other active control groups. For studies reporting follow-up data, analysis of the stability of treatment effects indicated that changes were stable over time (analyses are available in Multimedia Appendices 6, 7, and 8, respectively).

#### Web-Based Mindfulness Interventions and Quality of Life and Functioning Outcomes

**Figure 4:**
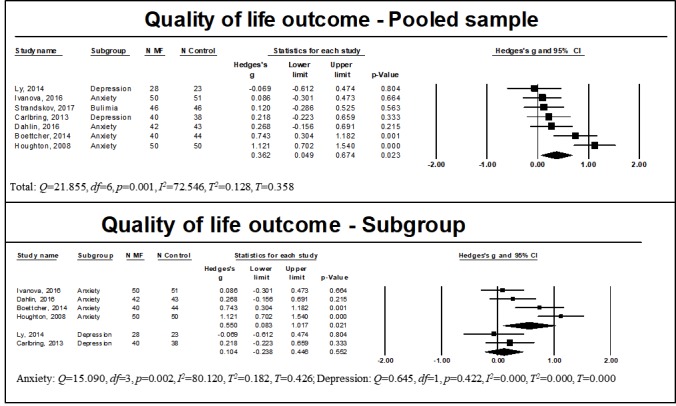
Results of quality of life outcome for the pooled and subgroup samples. MF: mindfulness intervention group.

Quality of life and functioning were examined by 10 studies, of which 4 included participants with anxiety disorders [36,52,56-59,61], 2 included participants with depressive disorders [37,53] and 1 included participants with bipolar disorder [55]. Usable data for meta-analysis of studies with control groups could be retrieved for 7 of them [36,37,52,53,56,59,61]. A summary of effect sizes is showed in Figure 4. We found a significant effect of WMIs on quality of life and functioning for the pooled sample (n=591, *g*=0.362, 95% CI 0.049 to 0.674, *P*=.02). Heterogeneity was noted (*Q*=21.855, *df*=6, *P*=.001, *I*^2^=72.546, *T*^2^=0.128, *T*=0.358). Subgroup analysis indicated a significant, moderate effect of WMIs on quality of life and functioning for those with a diagnosis of anxiety (n=370, *g*=0.550, 95% CI 0.083 to 1.017, *P*=.02), and heterogeneity was noted (*Q*=15.090, *df*=3, *P*=.002, *I*^2^=80.120, *T*^2^=0.182, *T*=0.426). However, there was no significant effect for those with a diagnosis of depression (n=129, *g*=0.104, 95% CI −0.238 to −0.446, *P*=.55), with no evidence of significant heterogeneity (*Q*=0.645, *df*=1, *P*=.42, *I*^2^=0.000, *T*^2^=0.000, *T*=0.000).

We performed subgroup analysis to examine differences between studies including mindfulness-only therapies [36,37,52] and those including mindfulness integrative therapies [53,56,59,61]. Subgroup analysis indicated that neither mindfulness integrative therapies, nor mindfulness-only therapies had a significant effect on quality of life and functioning. Studies investigating mindfulness integrative therapies showed no heterogeneity, while mindfulness-only therapies showed heterogeneity, which could be explained by baseline diagnosis (ie, anxiety vs depression) [36,37,52]. Furthermore, we examined differences between studies by type of control group. We did not find a significant effect of MBI on quality of life and functioning when compared to wait list or other active control groups. For studies reporting follow-up data, analysis of the stability of treatment effects indicated that changes were stable over time (analyses are available in Multimedia Appendices 9, 10, and 11, respectively).

**Figure 5:**
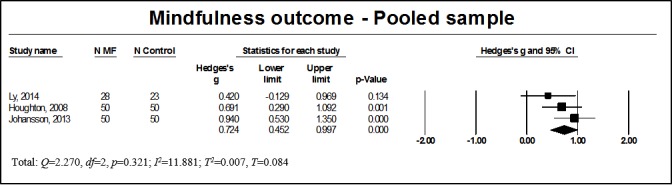
Results of mindfulness outcome for the pooled sample. MF: mindfulness intervention group.

#### Web-Based Mindfulness Interventions and Mindfulness Skills Outcomes

Mindfulness skills were examined in 4 studies, of which 3 included participants with anxiety disorders [52,57,60] and 2 included participants with depressive disorders [37,60]. Usable data for meta-analysis could be retrieved only for 3 of these [37,52,60]. A summary of effect sizes is shown in Figure 5. We found a significant large effect of WMIs on mindfulness skills for the pooled sample (n=251, *g*=0.724, 95% CI 0.452 to 0.997, *P*<.001). No heterogeneity was noted (*Q*=2.270, *df*=2, *P*=.32, *I*^2^=11.881, *T*^2^=0.007, *T*=0.084). Subgroup analysis could not be calculated due to the small sample size and heterogeneity of participants’ diagnoses. For studies reporting follow-up data, analysis of the stability of treatment effects indicated that changes were stable over time (all analyses are available in Multimedia Appendix 12).

### Publication Bias

Analysis of the funnel plot indicated some evidence of publication bias for the anxiety outcome. No publication bias was found for depression, quality of life and functioning, or mindfulness skills (Multimedia Appendices 13, 14, 15, and 16, respectively).

## Discussion

### Overview

The aim of this systematic review and meta-analysis was to examine the clinical and psychosocial effects of WMIs in patients with diagnosed mental health disorders. Overall, our results indicated that WMIs effectively reduced depression and anxiety symptoms and increased quality of life and functioning and mindfulness skills. The secondary aim of this study was to explore factors that can moderate the effects of WMIs in this population. In this respect, preliminary analyses provided initial evidence that WMIs may be particularly beneficial in patients with anxiety disorders, that mindfulness integrative online therapies may be more effective than mindfulness-only therapies and that WMIs may not be more effective than active control interventions.

### Effects of Mindfulness-Based Online Interventions on Depressive Symptoms

In relation to our primary aim, meta-analysis showed that WMIs reduced depressive symptoms in patients diagnosed with mental health conditions. However, further secondary analysis indicated that reduction of depressive symptoms was significant only for patients diagnosed with an anxiety disorder, and not for those with a diagnosis of depression. These findings are consistent with the results of a previous meta-analysis, which found a significant reduction in depressive symptoms associated with face-to-face MBCT in mental health patients with depression, anxiety, and bipolar disorder, when all were studied together [18]. Furthermore, our results are in keeping with Vøllestad et al’s [9] meta-analysis of face-to-face mindfulness interventions in patients diagnosed with anxiety, in which the researchers found a large reduction in comorbid depression symptoms associated with the use of mindfulness interventions. One possible explanation for these results could be that reduction of anxiety symptoms in those with a primary diagnosis of anxiety leads to reduced associated depressive symptoms. Alternatively, the low number of studies and statistical heterogeneity might explain the nonsignificant reduction of depressive symptoms in the depression subgroup, especially since the *P* value approaches significance.

Initial research showed that MBCT was effective only in those with >3 prior depressive episodes [19]. However, more recent studies have questioned these findings, indicating that MBCT may also be effective in those with one or two previous depressive episodes [62]. Unfortunately, with the exception of one study [37], we were unable to determine whether the study participants were experiencing their first episode of depression or had experienced recurrent depressive episodes. Thus, we could not perform a subgroup analysis to examine the differential effect of WMIs as a function of number of previous episodes. Future studies should examine the differential effectiveness of WMIs for different stages of illness.

In relation to the secondary analyses undertaken, results indicated that studies that used mindfulness integrative therapies reported significant reductions in depressive symptoms, while those that used mindfulness-only therapies did not. Although this finding indicates that implementing mindfulness techniques alongside other therapies may be more effective in reducing depressive symptoms than mindfulness alone, the low number of studies investigating mindfulness-only therapies requires that we interpret this result with caution.

In relation to the secondary finding that WMIs may have a significant effect in reducing depression when compared to wait list, but not when compared to active control groups, perhaps WMIs are not inferior to other active interventions in depression. Alternatively, this finding may be explained by the significant heterogeneity in the interventions and control groups used. One possible explanation may be that using a wait-list control may have a nocebo effect [63]. That is, using this control condition may result in detrimental effects in the control group and consequently larger effect sizes when compared to the treatment condition. Another possibility is that the digital placebo effect, by which digital interventions have increased effect sizes due to participants’ investment in the intervention [64], resulted in inflated effect sizes when an online intervention was compared with an offline control.

### Effects of Mindfulness-Based Online Interventions on Anxiety Symptoms

WMIs effectively reduced anxiety symptoms for the overall sample. However, secondary analyses indicated that this reduction was significant only for patients diagnosed with an anxiety disorder, and not for those diagnosed with depression. A recent meta-analysis has reported inconsistent findings on the effects of WMIs on anxiety, with effect sizes varying from small to large [32]. In contrast to our results, Strauss et al [8] found that face-to-face MBI did not reduce anxiety symptom severity among patients with an anxiety disorder. A larger meta-analysis that considered face-to-face mindfulness with patients diagnosed with anxiety disorders did find a large effect size [9]. The inconsistent results of these meta-analyses may be due to differing inclusion criteria, such as having a more inclusive definition of MBI [9] and using only group-based interventions [8], which may influence effects of mindfulness [23].

Secondary analyses indicated that WMIs applied to patients diagnosed with depression did not reduce anxiety symptoms. This may indicate that when anxiety symptoms are secondary to a primary diagnosis (in this case, depression), WMIs are less effective in reducing these symptoms. Alternatively, the low number of studies included could explain the nonsignificant reduction in anxiety symptoms in the depression subgroup results since the *P* value was approaching significance. Additional secondary analyses suggested that mindfulness integrative therapies might work better than mindfulness-only therapies, and WMIs appear to be more effective in the reduction of anxiety symptoms than a wait-list control (but not significantly inferior to other active conditions). As noted above, this may be explained by the heterogeneity in the intervention and control groups or the possibility of a nocebo or digital placebo effect influencing this result [63,64].

### Effect of Web-Based Mindfulness Interventions on Quality of Life and Functioning

WMIs significantly improved quality of life and functioning for the overall sample. However, secondary analyses indicated a significant improvement in quality of life and functioning for the anxiety disorder subgroup, but no improvement in the depressive disorder subgroup. Prior research has also shown varying results in relation to this outcome. Spijkerman, Pots, and Bohlmeijer [32] found a small effect size of WMIs on quality of life. In their meta-analysis, Vøllestad et al [9] found a large effect size when investigating the effect of face-to-face MBI on quality of life in patients with an anxiety disorder. Inconsistent findings may be explained by several variables, such as method of program delivery (ie, internet [32] or face-to-face [9]) or differences, such as diagnosis, between the samples of the trials included.

### Effect of Web-Based Mindfulness Interventions on Mindfulness Skills

We found a large effect of WMI on increasing mindfulness skills in the overall sample. The improvement in mindfulness skills found in our meta-analysis was consistent with Spijkerman et al’s [32] meta-analysis; however, we found a larger effect size. A possible reason is that our meta-analysis focused on a specific population of individuals diagnosed with a mental health condition, while Spijkerman et al’s [32] meta-analysis used broader inclusion criteria. In fact, Khoury, Lecomte, Gaudiano, and Paquin [17] have noted the importance of considering sample characteristics since MBIs have been found to be more effective in treating mental health conditions than physical or medical conditions.

### Strengths and Limitations

Our study is the first meta-analysis of WMIs focused on patients with diagnosed mental health conditions. We carefully assessed the quality of studies and mindfulness interventions, in line with recommendations by Higgins and Green [43] and Khoury et al [17]. Furthermore, inclusion of secondary analyses allowed for preliminary examination of variables that may impact the effectiveness of WMIs in this population.

A number of methodological issues should be considered for future research. The findings of this meta-analysis are limited by the small sample sizes of the studies included and the heterogeneity of WMIs. Specifically, the mindfulness interventions evaluated in the included studies varied in terms of the regularity of the program, whether homework was provided, and the extent of contact with therapist, among others. Given the low number of studies, we were unable to control for these variables. In addition, results for secondary analyses for anxiety and depression groups should be considered with caution, given the high comorbidity between these disorders [3]. Moreover, significant statistical heterogeneity in the active control condition subgroup limits our exploratory analysis of the relationship between type of control group and WMIs’ effectiveness. Notwithstanding these limitations, this meta-analysis provides initial evidence that WMIs can be an effective intervention for those with clinical anxiety and depression, with exploratory analyses indicating important areas for future research into variables that may moderate the impact of WMIs.

### Future Research

This meta-analysis revealed marked heterogeneity in the uptake and use of WMIs. This issue is of clinical relevance because research into online interventions has consistently demonstrated high attrition rates [65]. Future research should report what proportion of, and the degree to which, patients engage with different aspects of WMIs over time. Furthermore, noncompletion and good engagement should be measured according to a priori established criteria, and the design, content and interface aspects of WMIs should be carefully analyzed to study their potential differential effects. This will allow for the identification of variables that influence usage rates and treatment effects, and allow clinicians and researchers to tailor implementation of WMIs to maximize engagement and positive outcomes. This will facilitate a fuller understanding of what works well, and for whom. In addition, further studies should investigate the impact of WMIs in different mental health populations, as previous research has indicated the potential benefits of WMIs in young people at ultra-high risk of developing psychosis [66] and face-to-face MBI has been found effective for individuals with psychosis [67].

Currently, with the field of WMIs still in its early stages, the term WMI captures a broad, complex, and poorly defined class of interventions. Studies included in our meta-analysis did not clearly state the focus of each MBI (ie, to reduce stress, prevent depression relapse, or enhance well-being). In keeping with Crane et al’s [68] recommendations, future WMIs should follow clearly delineated protocols designed to ensure their quality and integrity. As such, further studies should clearly describe the focus, characteristics and expected therapeutic mechanisms of WMIs. This will not only help to better investigate the efficacy of WMIs (by allowing for comparison of interventions with differing therapeutic goals) but also to examine whether WMIs are exerting their effects via hypothesized treatment mechanisms. Ultimately, this will help inform the adaptation of specific WMIs to meet the needs and preferences of defined clinical populations, as well as to enhance their therapeutic impact by targeting therapeutic mechanisms more specifically.

Finally, future research should determine the effects of theory-driven, targeted WMIs when compared with (1) interventions controlling for unspecific therapeutic factors (eg, attention control) and (2) active interventions targeting differential mechanisms. In order to account for potential nocebo or digital placebo effects, further studies should include either attention control or active control groups. This is further supported by emerging evidence that mindfulness interventions are not inferior to traditional interventions such as cognitive behavioral therapy in the treatment of clinical depression or anxiety [17,69].

### Conclusion

In conclusion, our results indicate that WMIs may be an effective treatment modality for patients with diagnosed mental health disorders. Future studies should evaluate the effects of clearly described, theory-driven, high-quality, and targeted WMIs in varied clinical populations via well designed and powered controlled trials. Finally, future research should determine patient as well as intervention variables that determine the take-up and therapeutic effects of WMIs.

## Appendix

Multimedia Appendix 1 Characteristics of the selected studies.

Multimedia Appendix 2 Characteristics of the interventions.

Multimedia Appendix 3 Depression. Type of therapy and component.

Multimedia Appendix 4 Depression. Type of control.

Multimedia Appendix 5 Depression. Pretreatment vs F-U data.

Multimedia Appendix 6 Anxiety. Type of therapy and component.

Multimedia Appendix 7 Anxiety. Type of control.

Multimedia Appendix 8 Anxiety. Pretreatment vs F-U data.

Multimedia Appendix 9 Quality of Life. Type of therapy and component.

Multimedia Appendix 10 Quality of life. Type of control.

Multimedia Appendix 11 Quality of life. Pretreatment vs F-U data.

Multimedia Appendix 12 Mindfulness. Pretreatment vs F-U data.

Multimedia Appendix 13 Publication bias of anxiety outcome.

Multimedia Appendix 14 Publication bias of depression outcome.

Multimedia Appendix 15 Publication bias of quality of life.

Multimedia Appendix 16 Publication bias of mindfulness outcome.

## Abbreviations

MBCT: Mindfulness-based Cognitive Therapy
MBI: Mindfulness-based interventions
MBSR: Mindfulness-based stress reduction
TAU: treatment as usual
WMIs: Web-based mindfulness interventions

## References

[1] Steel Z, Marnane C, Iranpour C, Chey T, Jackson JW, Patel V, Silove D (2014). The global prevalence of common mental disorders: a systematic review and meta-analysis 1980-2013. Int J Epidemiol.

[2] Kessler RC, Aguilar-Gaxiola S, Alonso J, Chatterji S, Lee S, Ormel J, Ustün TB, Wang PS (2009). The global burden of mental disorders: an update from the WHO World Mental Health (WMH) surveys. Epidemiol Psichiatr Soc.

[3] Kessler RC, Sampson NA, Berglund P, Gruber MJ, Al-Hamzawi A, Andrade L, Bunting B, Demyttenaere K, Florescu S, de GG, Gureje O, He Y, Hu C, Huang Y, Karam E, Kovess-Masfety V, Lee S, Levinson D, Medina MME, Moskalewicz J, Nakamura Y, Navarro-Mateu F, Browne MAO, Piazza M, Posada-Villa J, Slade T, Ten HM, Torres Y, Vilagut G, Xavier M, Zarkov Z, Shahly V, Wilcox MA (2015). Anxious and non-anxious major depressive disorder in the World Health Organization World Mental Health Surveys. Epidemiol Psychiatr Sci.

[4] Bebbington P, Brugha T, Coid J, Crawford M, Deverill C, D Souza J (2009). Adult psychiatric morbidity in England Results of a household survey.

[5] McDermott B, Baigent M, Chanen A, Fraser L, Graetz B, Hayman N (2010). Clinical practice guidelines: depression in adolescents and young adults Internet.

[6] National Institute for Health and Care Excellence (NICE) (2011). Generalised anxiety disorder and panic disorder in adults: management Internet (Clinical guideline [CG113]).

[7] NICE (2010). Depression: The Nice Guideline on the Treatment and Management of Depression in Adults.

[8] Strauss C, Cavanagh K, Oliver A, Pettman D (2014). Mindfulness-based interventions for people diagnosed with a current episode of an anxiety or depressive disorder: a meta-analysis of randomised controlled trials. PLoS One.

[9] Vøllestad J, Nielsen MB, Nielsen GH (2012). Mindfulness- and acceptance-based interventions for anxiety disorders: a systematic review and meta-analysis. Br J Clin Psychol.

[10] NICE (2009). Depression: the treatment and management of depression in adults.

[11] Kabat-Zinn J (1990). Full Catastrophe Living: Using the Wisdom of your Body and Mind to Face Stress, Pain and Illness.

[12] Hayes S, Strosahl K (2004). A Practical Guide to Acceptance and Commitment Therapy.

[13] Williams JMG (2010). Mindfulness and psychological process. Emotion.

[14] Godfrin KA, van HC (2010). The effects of mindfulness-based cognitive therapy on recurrence of depressive episodes, mental health and quality of life: A randomized controlled study. Behav Res Ther.

[15] Teasdale JD, Segal ZV, Williams JM, Ridgeway VA, Soulsby JM, Lau MA (2000). Prevention of relapse/recurrence in major depression by mindfulness-based cognitive therapy. J Consult Clin Psychol.

[16] Hayes S, Strosahl K, Wilson K (1999). Acceptance and Commitment Therapy: An experiential approach to behavior change.

[17] Khoury B, Lecomte T, Fortin G, Masse M, Therien P, Bouchard V, Chapleau M, Paquin K, Hofmann SG (2013). Mindfulness-based therapy: a comprehensive meta-analysis. Clin Psychol Rev.

[18] Chiesa A, Serretti A (2011). Mindfulness based cognitive therapy for psychiatric disorders: a systematic review and meta-analysis. Psychiatry Res.

[19] Piet J, Hougaard E (2011). The effect of mindfulness-based cognitive therapy for prevention of relapse in recurrent major depressive disorder: A systematic review and meta-analysis. Clin Psychol Rev.

[20] Kuyken W, Warren FC, Taylor RS, Whalley B, Crane C, Bondolfi G, Hayes R, Huijbers M, Ma H, Schweizer S, Segal Z, Speckens A, Teasdale JD, Van HK, Williams M, Byford S, Byng R, Dalgleish T (2016). Efficacy of Mindfulness-Based Cognitive Therapy in Prevention of Depressive Relapse: An Individual Patient Data Meta-analysis From Randomized Trials. JAMA Psychiatry.

[21] Janssen L, Kan CC, Carpentier PJ, Sizoo B, Hepark S, Grutters J, Donders R, Buitelaar JK, Speckens AEM (2015). Mindfulness based cognitive therapy versus treatment as usual in adults with attention deficit hyperactivity disorder (ADHD). BMC Psychiatry.

[22] Chiesa A, Serretti A (2014). Are Mindfulness-Based Interventions Effective for Substance Use Disorders? A Systematic Review of the Evidence. Subst Use Misuse.

[23] Carmody J, Baer RA (2009). How long does a mindfulness-based stress reduction program need to be? A review of class contact hours and effect sizes for psychological distress. J Clin Psychol.

[24] McRoberts C, Burlingame G, Hoag M (1998). Comparative efficacy of individual and group psychotherapy: A meta-analytic perspective. Gr Dyn Theory Res Pract.

[25] Tucker M, Oei T (2007). Is Group More Cost Effective than Individual Cognitive Behaviour Therapy? The Evidence is not Solid Yet. Behav Cogn Psychother.

[26] Oh E, Jorm AF, Wright A (2009). Perceived helpfulness of websites for mental health information: a national survey of young Australians. Soc Psychiatry Psychiatr Epidemiol.

[27] Álvarez-Jiménez M, Gleeson JF, Bendall S, Lederman R, Wadley G, Killackey E, McGorry PD (2012). Internet-based interventions for psychosis: a sneak-peek into the future. Psychiatr Clin North Am.

[28] Wang PS, Simon G, Kessler RC (2003). The economic burden of depression and the cost-effectiveness of treatment. Int J Methods Psychiatr Res.

[29] Firth J, Torous J, Nicholas J, Carney R, Pratap A, Rosenbaum S, Sarris Jerome (2017). The efficacy of smartphone-based mental health interventions for depressive symptoms: a meta-analysis of randomized controlled trials. World Psychiatry.

[30] Firth J, Torous J, Nicholas J, Carney R, Rosenbaum S, Sarris J (2017). Can smartphone mental health interventions reduce symptoms of anxiety? A meta-analysis of randomized controlled trials. J Affect Disord.

[31] NICE (2006). Computerised cognitive behaviour therapy for depression and anxiety Internet (Technology appraisal guidance [TA97]).

[32] Spijkerman M, Pots W, Bohlmeijer E (2016). Effectiveness of online mindfulness-based interventions in improving mental health: A review and meta-analysis of randomised controlled trials. Clin Psychol Rev.

[33] Boggs Jennifer M, Beck Arne, Felder Jennifer N, Dimidjian Sona, Metcalf Christina A, Segal Zindel V (2014). Web-based intervention in mindfulness meditation for reducing residual depressive symptoms and relapse prophylaxis: a qualitative study. J Med Internet Res.

[34] Cavanagh K, Strauss C, Cicconi F, Griffiths N, Wyper A, Jones F (2013). A randomised controlled trial of a brief online mindfulness-based intervention. Behav Res Ther.

[35] Hesser H, Gustafsson T, Lundén C, Henrikson O, Fattahi K, Johnsson E, Zetterqvist WV, Carlbring P, Mäki-Torkko E, Kaldo V, Andersson G (2012). A randomized controlled trial of Internet-delivered cognitive behavior therapy and acceptance and commitment therapy in the treatment of tinnitus. J Consult Clin Psychol.

[36] Boettcher J, Aström V, Påhlsson D, Schenström O, Andersson G, Carlbring P (2014). Internet-based mindfulness treatment for anxiety disorders: a randomized controlled trial. Behav Ther.

[37] Ly K, Trüschel Anna, Jarl L, Magnusson S, Windahl T, Johansson R, Carlbring Per, Andersson Gerhard (2014). Behavioural activation versus mindfulness-based guided self-help treatment administered through a smartphone application: a randomised controlled trial. BMJ Open.

[38] Liberati A, Altman DG, Tetzlaff J, Mulrow C, Gøtzsche PC, Ioannidis JPA, Clarke M, Devereaux PJ, Kleijnen J, Moher D (2009). The PRISMA statement for reporting systematic reviews and meta-analyses of studies that evaluate healthcare interventions: explanation and elaboration. BMJ.

[39] American Psychiatry Association (APA) (2000). Diagnostic and Statistical Manual of Mental Disorders: DSM-IV-TR.

[40] World Health Organization (WHO) (1992). The ICD-10 classification of mental and behavioural disorders: Clinical descriptions and diagnostic guidelines.

[41] Shawyer F, Farhall J, Mackinnon A, Trauer T, Sims E, Ratcliff K, Larner C, Thomas N, Castle D, Mullen P, Copolov D (2012). A randomised controlled trial of acceptance-based cognitive behavioural therapy for command hallucinations in psychotic disorders. Behav Res Ther.

[42] Alvarez-Jimenez M, Alcazar-Corcoles MA, González-Blanch C, Bendall S, McGorry PD, Gleeson JF (2014). Online, social media and mobile technologies for psychosis treatment: a systematic review on novel user-led interventions. Schizophr Res.

[43] Higgins J, Green S (2011). Cochrane Handbook for Systematic Reviews of Interventions Version 5.1.0.

[44] Baer RA, Smith GT, Hopkins J, Krietemeyer J, Toney L (2006). Using self-report assessment methods to explore facets of mindfulness. Assessment.

[45] Bond FW, Hayes SC, Baer RA, Carpenter KM, Guenole N, Orcutt HK, Waltz T, Zettle RD (2011). Preliminary psychometric properties of the Acceptance and Action Questionnaire-II: a revised measure of psychological inflexibility and experiential avoidance. Behav Ther.

[46] Baum C, Kuyken W, Bohus M, Heidenreich T, Michalak J, Steil R (2010). The psychometric properties of the Kentucky Inventory of Mindfulness Skills in clinical populations. Assessment.

[47] Cardaciotto L, Herbert JD, Forman EM, Moitra E, Farrow V (2008). The assessment of present-moment awareness and acceptance: the Philadelphia Mindfulness Scale. Assessment.

[48] Borenstein M, Hedges LV, Higgins JPT, Rothstein HR (2006). Comprenhensive Meta-Analysis Software.

[49] Cohen J (1988). Statistical Power Analysis for the Behavioral Sciences.

[50] Cohen J (1992). A power primer. Psychol Bull.

[51] Borenstein M, Hedges LV, Higgins J, Rothstein H (2009). Introduction to Meta-Analysis.

[52] Houghton V (2008). A Quantitative Study of the Effectiveness of Mindfulness-Based Stress Reduction Treatment, Using an Internet-Delivered Self-Help Progran, for Women with Generalized Anxiety Disorder.

[53] Carlbring P, Hägglund M, Luthström A, Dahlin M, Kadowaki Å, Vernmark K (2013). Internet-based behavioral activation and acceptance-based treatment for depression: A randomized controlled trial Internet. J Affect Disord.?.

[54] Kivi M, Eriksson MCM, Hange D, Petersson E, Vernmark K, Johansson B, Björkelund C (2014). Internet-based therapy for mild to moderate depression in Swedish primary care: short term results from the PRIM-NET randomized controlled trial. Cogn Behav Ther.

[55] Murray G, Leitan N, Berk M, Thomas N, Michalak E, Berk L, Johnson S L, Jones S, Perich T, Allen N B, Kyrios M (2015). Online mindfulness-based intervention for late-stage bipolar disorder: pilot evidence for feasibility and effectiveness. J Affect Disord.

[56] Dahlin M, Andersson G, Magnusson K, Johansson T, Sjögren J, Håkansson A, Pettersson M, Kadowaki Å, Cuijpers P, Carlbring P (2016). Internet-delivered acceptance-based behaviour therapy for generalized anxiety disorder: A randomized controlled trial. Behav Res Ther.

[57] Gershkovich M, Herbert J, Forman E, Glassman L (2016). Guided Internet-Based Self-Help Intervention for Social Anxiety Disorder With Videoconferenced Therapist Support. Cognitive and Behavioral Practice.

[58] Carlbring P, Hägglund M, Luthström A, Dahlin M, Kadowaki Å, Vernmark K (2013). Internet-based behavioral activation and acceptance-based treatment for depression: A randomized controlled trial Internet. J Affect Disord.?.

[59] Ivanova E, Lindner P, Ly KH, Dahlin M, Vernmark K, Andersson G, Carlbring P (2016). Guided and unguided Acceptance and Commitment Therapy for social anxiety disorder and/or panic disorder provided via the Internet and a smartphone application: A randomized controlled trial. J Anxiety Disord.

[60] Johansson R, Björklund M, Hornborg C, Karlsson S, Hesser H, Ljótsson B, Rousseau A, Frederick RJ, Andersson G (2013). Affect-focused psychodynamic psychotherapy for depression and anxiety through the Internet: a randomized controlled trial. PeerJ.

[61] Strandskov SW, Ghaderi A, Andersson H, Parmskog N, Hjort E, Wärn AS, Jannert M, Andersson G (2017). Effects of Tailored and ACT-Influenced Internet-Based CBT for Eating Disorders and the Relation Between Knowledge Acquisition and Outcome: A Randomized Controlled Trial. Behav Ther.

[62] Geschwind N, Peeters F, Huibers M, van OJ, Wichers M (2012). Efficacy of mindfulness-based cognitive therapy in relation to prior history of depression: randomised controlled trial. Br J Psychiatry.

[63] Furukawa TA, Noma H, Caldwell DM, Honyashiki M, Shinohara K, Imai H, Chen P, Hunot V, Churchill R (2014). Waiting list may be a nocebo condition in psychotherapy trials: a contribution from network meta-analysis. Acta Psychiatr Scand.

[64] Torous J, Firth J (2016). The digital placebo effect: mobile mental health meets clinical psychiatry. Lancet Psychiatry.

[65] Eysenbach G (2005). The law of attrition. J Med Internet Res.

[66] Alvarez-Jimenez M, Gleeson J, Bendall S, Rice S, D Alfonso S, Eleftheriadis D, Russon P, Rumney L, Mackinnon J, Nelson B (2017). SU125. Momentum: A Novel Online Social Media, Mindfulness, and Strengths-Based Intervention to Promote Functional Recovery in Ultra High Risk (UHR) Patients. Schizophr Bull.

[67] Khoury B, Lecomte T, Gaudiano BA, Paquin K (2013). Mindfulness interventions for psychosis: a meta-analysis. Schizophr Res.

[68] Crane RS, Brewer J, Feldman C, Kabat-Zinn J, Santorelli S, Williams JMG, Kuyken W (2017). What defines mindfulness-based programs? The warp and the weft. Psychol Med.

[69] Chiesa A, Castagner V, Andrisano C, Serretti A, Mandelli L, Porcelli S, Giommi F (2015). Mindfulness-based cognitive therapy vs. psycho-education for patients with major depression who did not achieve remission following antidepressant treatment. Psychiatry Res.

