# Supplementary material for: Web-Based Mindfulness Interventions for Mental Health Treatment: Systematic Review and Meta-Analysis
Source: JMIR Ment Health. 2018 Sep 25;5(3):e10278. doi: 10.2196/10278 (PMC6231788; doi:10.2196/10278)
Supplement: Multimedia Appendix 1 [file mental_v5i3e10278_app1.pdf]

Table 1. Characteristics of the selected studies.

Study	Diagnosis and criteria	N	% female	Mean age in years (SD)	Length of treatment in weeks	F-U[a] months	Control group (n)	Length of control (weeks)	Primary outcomes
Boettcher et al, 2014	Anxiety disorders. SCID[b]	45	76	37.9 (8.9)	8	6	Discussion forum (46)	8	Anxiety
Ly et al, 2016	Major depression. MINI[c]	28	70	36 (10.8)	8	6	Behavioral activation (23)	8	Depression
Carlbring et al, 2013	Major depressive episode. SCID	40	83	44.4 (13.5)	8	3	Wait list (40)	8	Depression
Kivi et al, 2014	Depression disorder. MINI and score less than 35 on the MADRS-S[d]	36	66	36.6 (11.3)	12	3	TAU[e] (43)	12	Depression
Murrayet al, 2015	Bipolar disorder. Clinician criteria	16	N/A[f]	46.6 (12.9)	3	N/A	N/A	N/A	Quality of life
Dahlin et al, 2016	Generalized anxiety disorder. SCID	52	84	39.48 (10.73)	9	6	Wait list (51)	9	Anxiety
Gershkovich et al, 2016	Social anxiety disorder. MINI and SCID	13	69	33.2 (10.4)	8	3	N/A	N/A	Anxiety
Gershkovich et al, 2017	Social Anxiety disorder. MINI and Social anxiety disorder section of the SCID and ADIS[g]	42	64	31.5 (9.95)	8	N/A	N/A	N/A	Anxiety
Houghton 2008	Generalized anxiety disorder. Clinician criteria	50	100	43 (8.9)	8	N/A	Wait list (50)	8	Anxiety
Ivanova et al 2016	Social anxiety disorder and Panic Disorder. SCID	50	60	35.29 (10.98)	10	12	Wait list (51)	10	Anxiety
Johansson et al, 2013	Depressive and anxiety disorders. MINI	28 and 22	82	44 (13.1)	10	7	Empathetic listening (29 and 21)	10	Anxiety and Depression
Strandskov et al, 2017	Bulimia nerviosa and Eating disorders not otherwise specified. MINI	46	100	29.14 (9.69)	8	N/A	Wait list (46)	8	Eating disorder psychopath ology

[a]F-U months: Number of months between baseline and follow-up assessments.

[b]SCID: Structured Clinical Interview for Diagnostic and Statistical Manual of Mental Disorders.

[c]MINI: International Neuropsychiatric Interview.

[d]MADRS-S: Montgomery–Åsberg Depression Rating Scale.

[e]TAU: Treatment as usual.

[f]N/A: Not applicable.

[g]ADIS: Anxiety Disorders Interview schedule.
