# Supplementary material for: Web-Based Mindfulness Interventions for Mental Health Treatment: Systematic Review and Meta-Analysis
Source: JMIR Ment Health. 2018 Sep 25;5(3):e10278. doi: 10.2196/10278 (PMC6231788; doi:10.2196/10278)
Supplement: Multimedia Appendix 2 [file mental_v5i3e10278_app2.pdf]

Table 2. Characteristics of the interventions.

Study	Type of intervention	Length of treatment in weeks	Material used	Regularity	Setting	Implementation structure	Assigned home task(s)	Contact with therapist
Boettcher et al, 2014	Mindfulness	8	Videos or audio files or written instructions	6 out of 7	C-B[a]	8 M access to the next M once the previous in completed	Yes. 2 per day, every day (20 min for both tasks)	Email encouraging participants to continue sent in week 4
Ly et al, 2014	Mindfulness	8	Text psychoeducation or audio tracks	Flexible- Encouraged to log in daily	S-P[b]	Not structured	Yes. Write a reflection weekly	Encouraging short message service text messages sent every other day and general educational message sent weekly via email
Carlbring et al, 2013	BA[c]+ACT[d]	8	Written text or videos or narrated animations. Workbook on paper and CD[e]	Flexible- Log in once a week recommended	C-B	7 M, all available, order recommended but not enforced	Yes. Paper workbook homework each M[f]	Email with coach intervention
Kivi et al, 2014	BA+ACT	12	Written text or videos or audio files- workbook on paper and CD room (apart from online content)	Flexible	C-B	7 M, all available, order recommended but not enforced	Yes. Paper workbook homework each M	Email or phone with coach intervention
Murray et al, 2015	ACT+MBCT[g]	3	Video or audio or handouts	Flexible	C-B	3 M-all available	Yes	N/A[h]
Dahlinet al, 2016	Acceptance-based behavior therapy	9	Text, audio, animation, video + workbook on paper and audio CD	Flexible	C-B	7 M, all available, order recommended but not enforced	Yes	Secure short message service text messaging with coach interventions and clarify problems with the intervention
Gershkovich et al, 2016	Acceptance-based CBT[i]	8	Video clips or quizzes or slides w audio narration	Flexible	C-B	8 M - 1 p/w[j]	Yes- depends on M (reading, exposure…)	Videoconference with coach intervention, clarify treatment conceptions, address technological questions. Email to encourage the completion of M
Gershkovich et al, 2017	Acceptance-based CBT	8	Video clips or quizzes or slides with audio narration	Flexible	C-B	8 M - 1 p/w	Yes- depends on M (reading, exposure…)	Half the sample had weekly videoconferences with therapist (e-coach) and received daily short message service text messages
Houghton	MBSR[k]	8	n/r[l]	6-7 out of 7	C-B	8M - 1 p/w	n/r	n/r

2008								
Ivanova et al, 2016	ACT	10	Written text or videos or narrated animations. Workbook on paper and CD. Smartphone app	n/r	C-B and S-P	8M	Yes. Paper workbook homework each M	Half the sample received feedback on their treatment from a therapist and the other half received automated feedback
Johansson et al, 2013	AFPP[m]	10	Written text, figures, audio files	Flexible	C-B	8 M	Yes	Feedback of the M
Strandskov et al, 2017	ACT-influenced internet-based CBT	8	Interactive material, exercises	Daily	C-B	8M - 1 p/w	Yes	Short feedback on homework, reminders, and support via website. Telephone contact if initiated by therapist

[a]C-B: Computer-based.

[b]S-P: Smartphone.

[c]BA: Behavioral activation.

[d]ACT: Acceptance and Commitment Therapy.

[e]CD: Compact disc.

[f]M: Modules.

[g]MBCT: Mindfulness-based cognitive therapy.

[h]N/A: not applicable.

[i]CBT: Cognitive behavioral therapy.

[j]p/w: per week.

[k]MBSR: Mindfulness-based stress reduction.

[l]n/r: not reported.

[m]AFPP: Affect-focused psychodynamic psychotherapy.
