# Supplementary figures and images for: Web-Based Mindfulness Interventions for Mental Health Treatment: Systematic Review and Meta-Analysis
Source: JMIR Ment Health. 2018 Sep 25;5(3):e10278. doi: 10.2196/10278 (PMC6231788; doi:10.2196/10278)

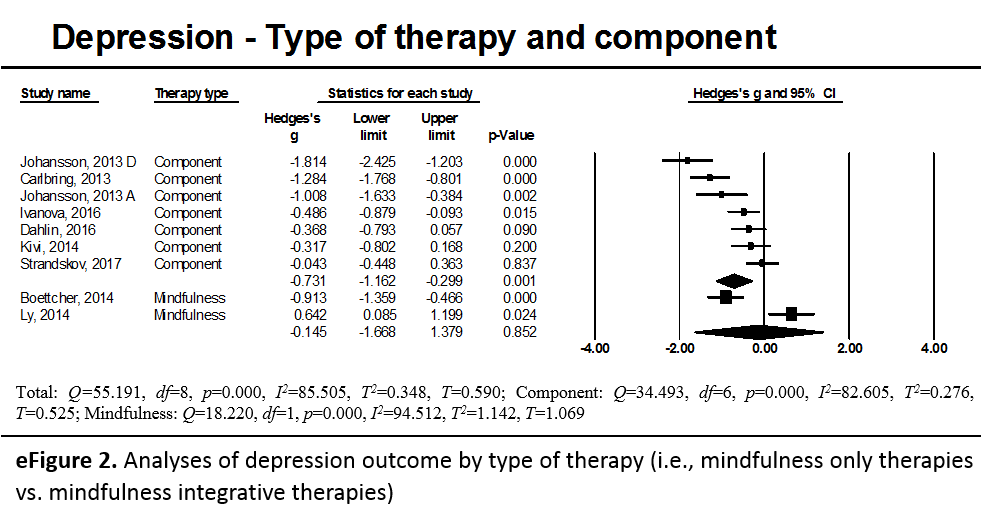

Supplement: Multimedia Appendix 3 [file mental_v5i3e10278_app3.png]

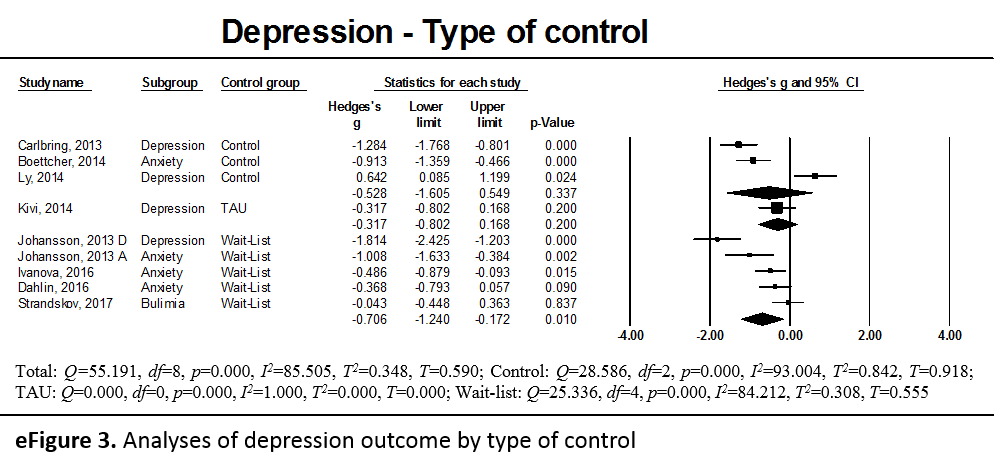

Supplement: Multimedia Appendix 4 [file mental_v5i3e10278_app4.png]

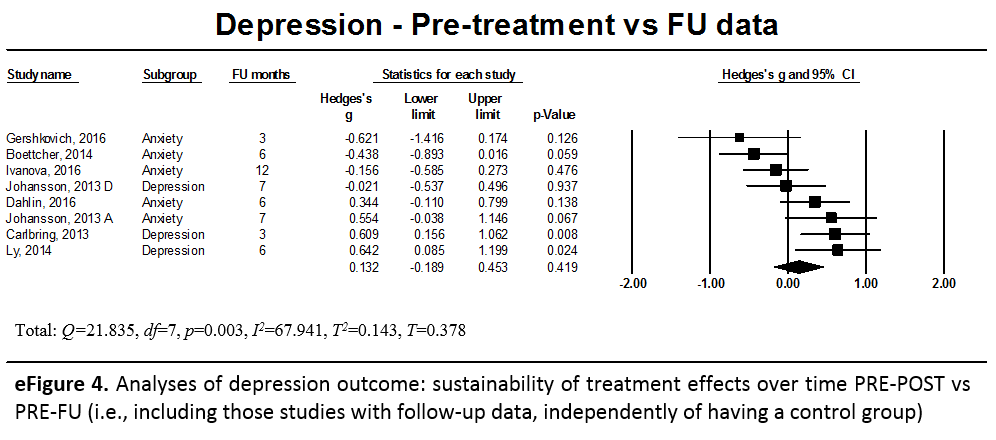

Supplement: Multimedia Appendix 5 [file mental_v5i3e10278_app5.png]

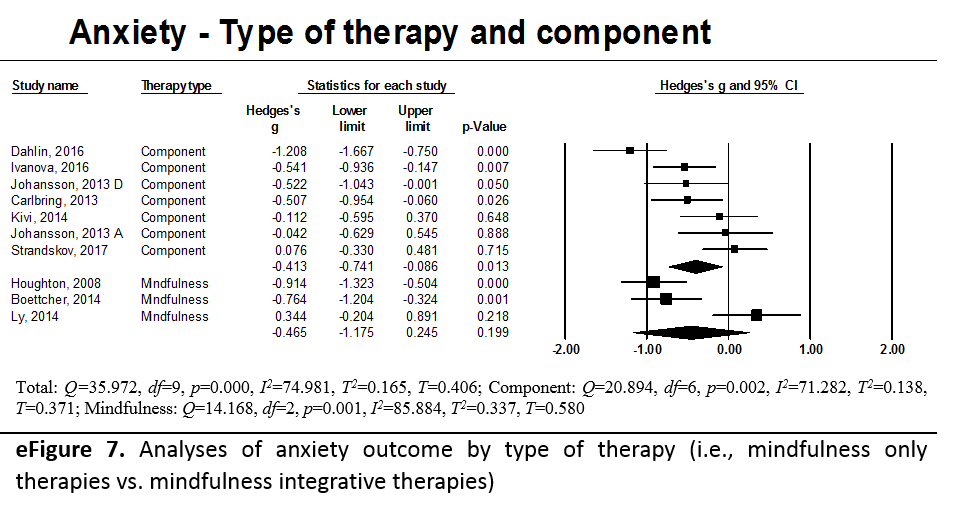

Supplement: Multimedia Appendix 6 [file mental_v5i3e10278_app6.png]

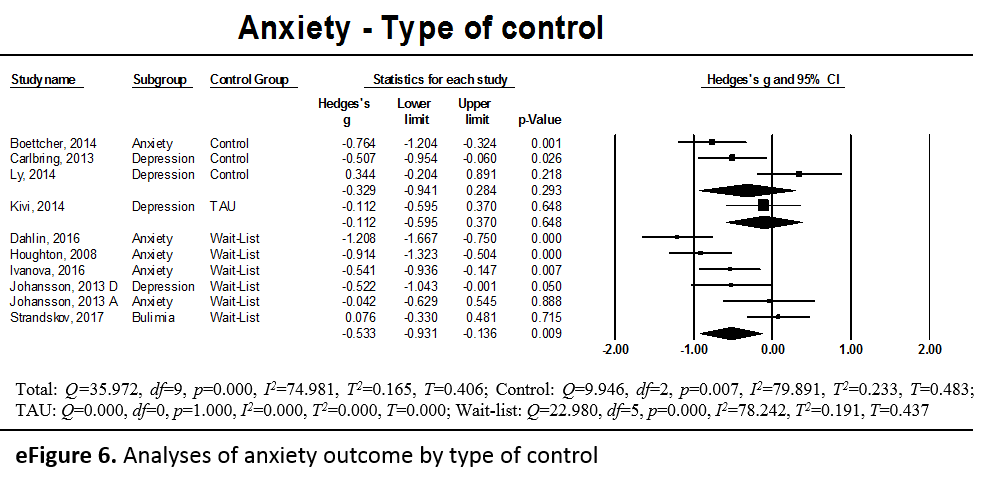

Supplement: Multimedia Appendix 7 [file mental_v5i3e10278_app7.png]

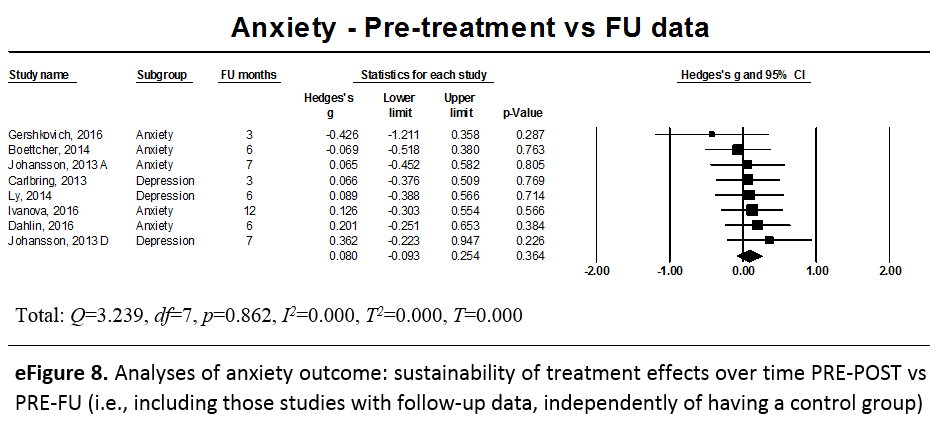

Supplement: Multimedia Appendix 8 [file mental_v5i3e10278_app8.png]

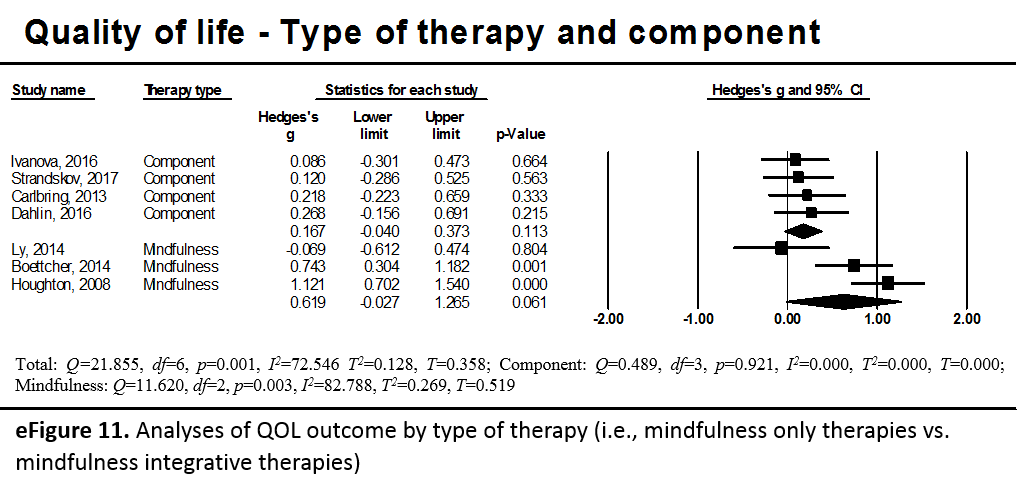

Supplement: Multimedia Appendix 9 [file mental_v5i3e10278_app9.png]

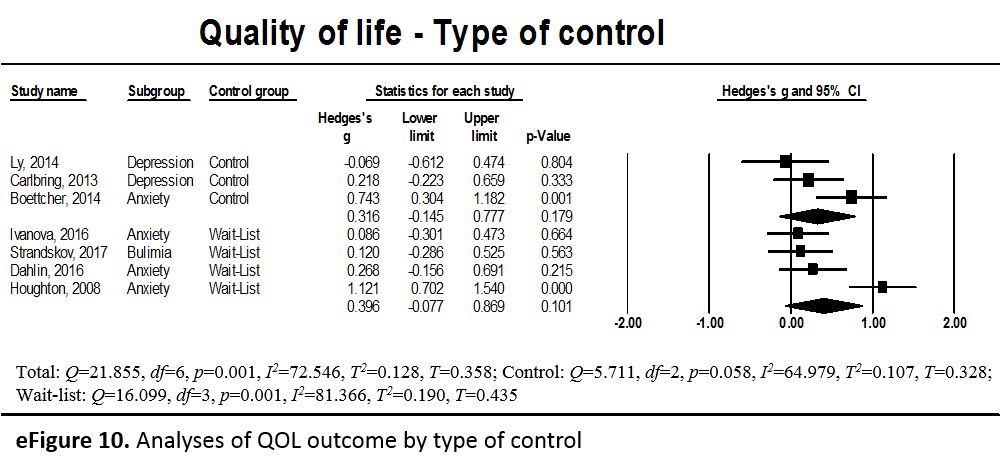

Supplement: Multimedia Appendix 10 [file mental_v5i3e10278_app10.png]

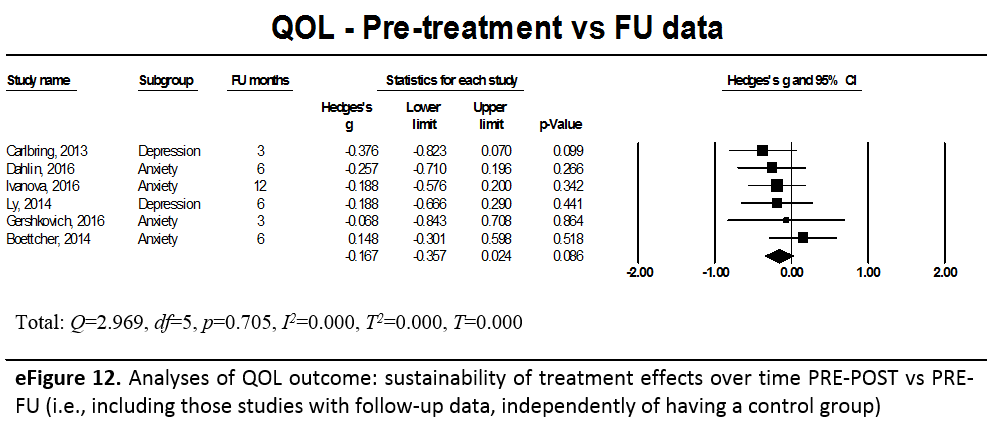

Supplement: Multimedia Appendix 11 [file mental_v5i3e10278_app11.png]

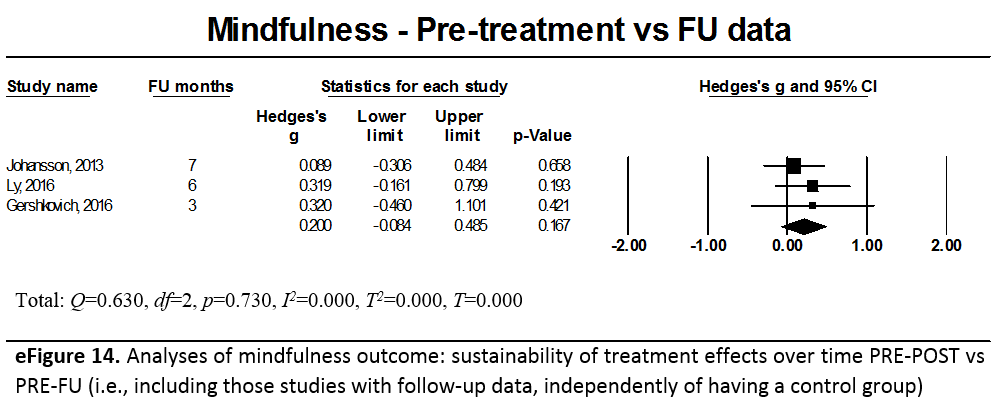

Supplement: Multimedia Appendix 12 [file mental_v5i3e10278_app12.png]

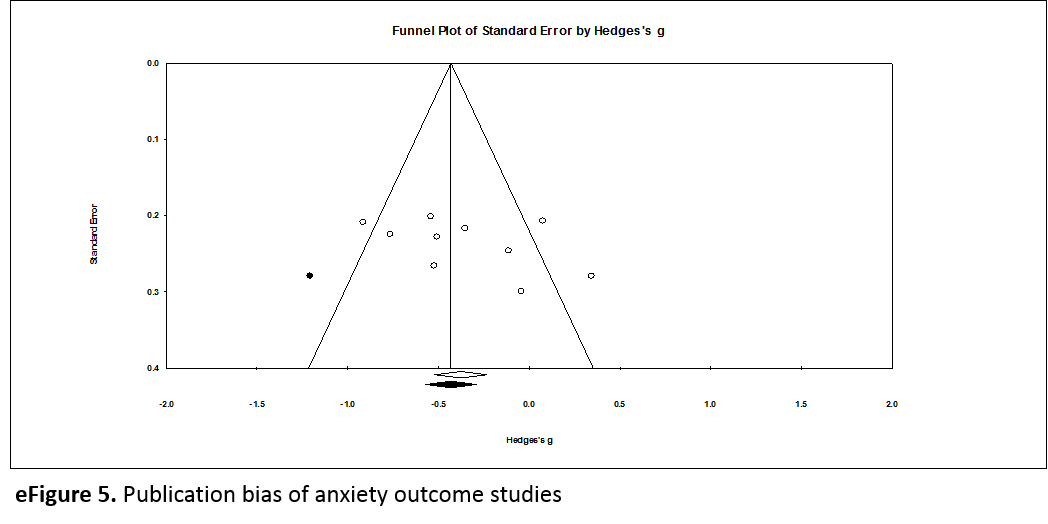

Supplement: Multimedia Appendix 13 [file mental_v5i3e10278_app13.png]

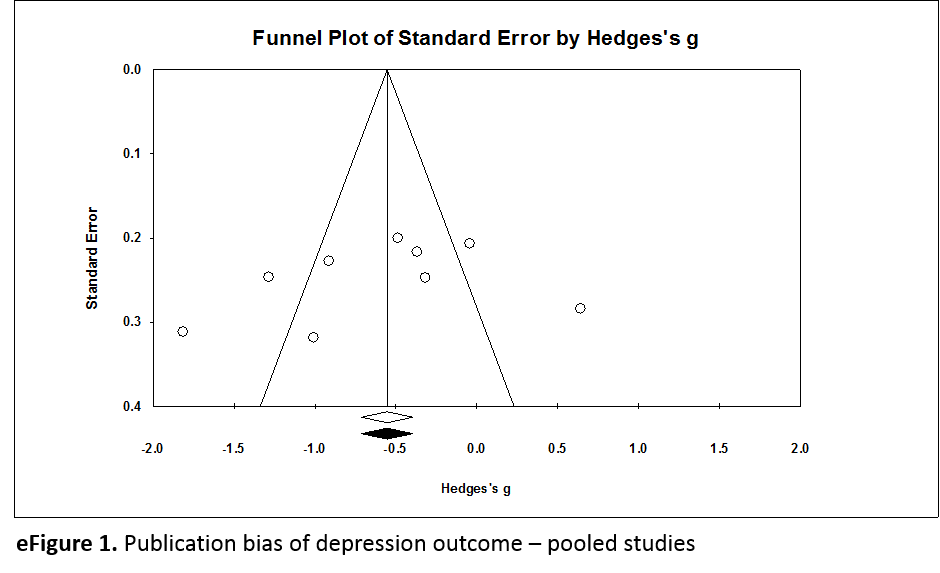

Supplement: Multimedia Appendix 14 [file mental_v5i3e10278_app14.png]

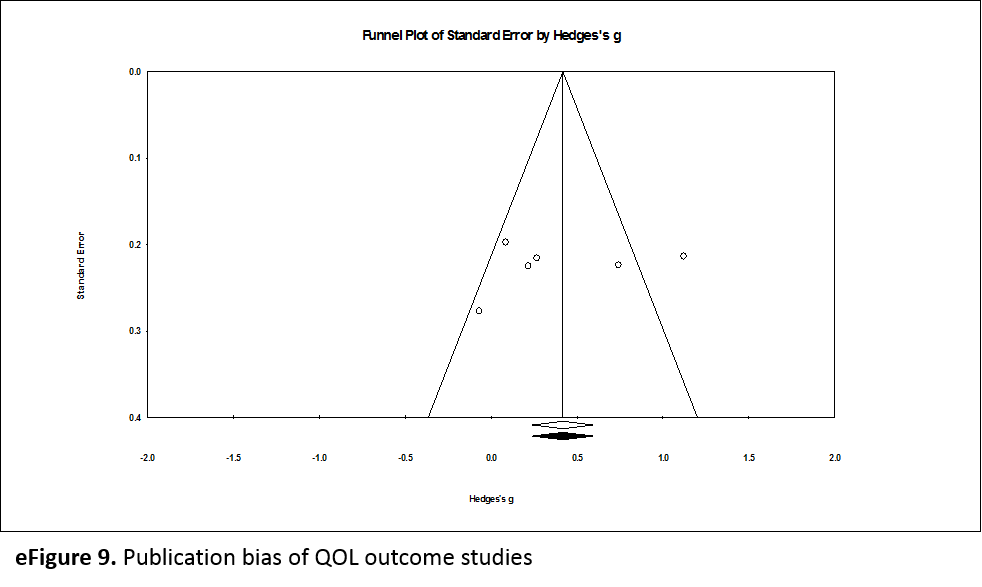

Supplement: Multimedia Appendix 15 [file mental_v5i3e10278_app15.png]

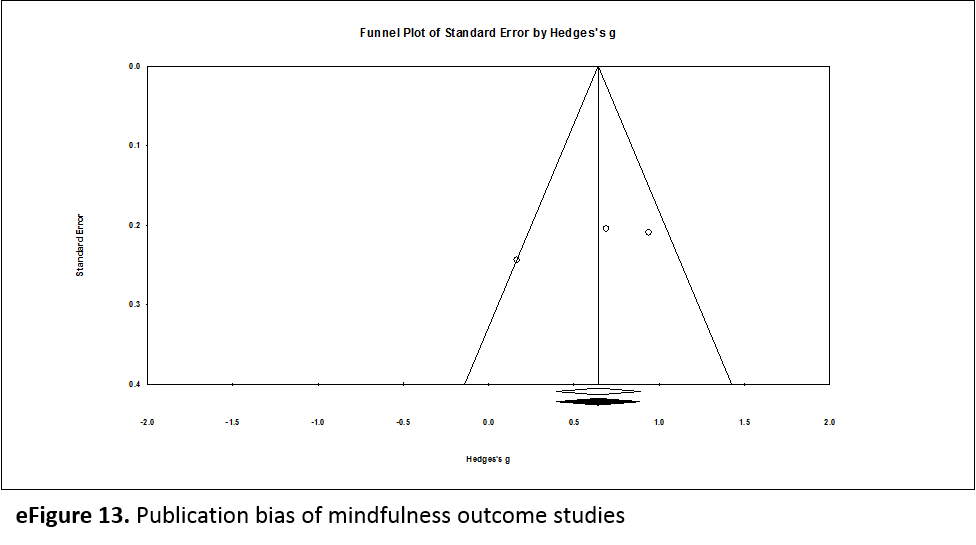

Supplement: Multimedia Appendix 16 [file mental_v5i3e10278_app16.png]
